# Gross Motor Skills Are Associated with Symptoms of Attention Deficit Hyperactivity Disorder in School-Aged Children

**DOI:** 10.3390/children11070757

**Published:** 2024-06-21

**Authors:** Cristiana D’Anna, Fabio Carlevaro, Francesca Magno, Roberto Vagnetti, Pierpaolo Limone, Daniele Magistro

**Affiliations:** 1 Department of Psychology and Education, Pegaso University, 80143 Napoli, Italy; pierpaolo.limone@unipegaso.it; 2 Polo Universitario Asti Studi Superiori (Uni-Astiss), 14100 Asti, Italy; 3 Dipartimento di Scienze della Vita e Biologia dei Sistemi, University of Torino, 10124 Torino, Italy; 4 Department of Sport Science, School of Science and Technology, Nottingham Trent University, Nottingham NG1 4FQ, UK

**Keywords:** attention deficit hyperactivity disorder, development, gross motor skills, inattention, impulsivity, motor competence

## Abstract

Attention deficit hyperactivity disorder (ADHD) is among the most prevalent disorders in children and is frequently linked with motor difficulties that can impact both daily motor tasks and overall developmental trajectories. The objective of this study was to analyse the association between gross motor skills and ADHD symptoms. Using a cross-sectional research design, data were collected from a sample of primary school children (N = 2677; mean age = 8.58 years, SD = 1.49 years). The Gross Motor Development-3 Test (TGMD-3) was employed to assess participants’ gross motor skills, whereas the ADHD Rating Scale (SDAI), completed by teachers, evaluated ADHD symptoms through two subscales: inattention and impulsivity/hyperactivity. The results revealed an association between motor development and ADHD symptoms, with greater proficiency in gross motor skills correlating with lower symptoms reported on the SDAI. Logistic regression analyses indicated that the TGMD-3 was significantly associated with the risk of ADHD in matched samples of at-risk children and controls. The evaluation of gross motor development proves to be a useful tool for monitoring global development, paying attention to any critical issues, particularly in relation to the variables of inattention and hyperactivity.

## 1. Introduction

Attention deficit hyperactivity disorder (ADHD) is one of the most common disorders in children [1] and is often associated with motor difficulties, difficulty controlling behaviour, and impairments in executive functions [2].

According to the American Psychiatric Association [3,4], ADHD is a neurodevelopmental disorder defined by impaired levels of inattention, disorganization, and/or hyperactivity/impulsivity that are persistent, maladaptive, and inconsistent with their developmental level. ADHD is diagnosed clinically following multidisciplinary assessments, which include medical, developmental, educational, and comprehensive psychological evaluations. The DSM-5-TR [4] diagnostic criteria list nine symptoms of inattention and of hyperactivity and impulsivity. These symptoms must have been present for at least 6 months, be more pronounced than expected for a child at a comparable developmental stage, occur in at least two different settings, manifest before the age of 12, and interfere with daily routines at school and within the family. Three subtypes of ADHD have been recognised, which include a predominantly inattentive presentation, a predominantly hyperactive/impulsive presentation, and a combined presentation of symptoms [4].

The aforementioned characteristics of the disorder inevitably compromise not only the child’s motor development but also affect all domains of their personality and, in general, lead to a lower quality of life with compromised social relationships with peers [5,6]. Children with ADHD who struggle with motor skills often face challenges completing tasks that require coordinated movements [7,8]. Importantly, the difficulties experienced by these individuals have a strong impact on their academic and professional success [5,9].

There is evidence suggesting that executive difficulties may be related to the disorder, and importantly, these skills also encompass sensory and motor components [10]. The inattention and lack of inhibition that characterise the disorder could explain the difficulties in fine and gross motor skills evidenced in the literature, which in some cases could be severe [11]. A recent meta-analysis indicated a positive relationship between motor skills and executive functions in children [12], confirming the notion that both are served by overlapping networks [13]. However, for ADHD, it has been proposed that typical symptoms may be related to dopamine circuits [14], where a hypofunctioning nigrostriatal dopaminergic pathway could cause impaired control of motor skills, and a hypofunctioning mesocortical dopamine pathway could be related to poor executive functions [15].

Kaiser et al. (2015) found in a review that the majority of children with ADHD exhibit poorer motor skills than their peers without ADHD, with an uneven profile of impairment across different motor tasks [16]. This can potentially influence the trajectory of their overall development. Two possible hypotheses persist to explain the relationship between motor difficulties and ADHD [17]: the first suggests that the link derives directly from the basic symptoms of ADHD, whereas the second suggests that motor difficulties derive from the coexistence of ADHD with developmental coordination disorder (DCD), a neurodevelopmental disorder characterised by fine and gross motor coordination difficulties, which leads to difficulties in the acquisition and execution of coordinated motor skills [3].

During development, children acquire fundamental skills and learn new motor patterns [18]. Their performance in motor and recreational activities influences how classmates perceive them, which, in turn, indirectly affects their motivation to participate in group activities, thereby limiting opportunities for interaction and experiencing new learning situations [19,20]. The worldwide prevalence of ADHD has been estimated at 5% [21], but more recent studies indicate that this has increased to 7.6% in children aged 3 to 12 years and 5.6% in teenagers aged 12 to 18 years [22], with a persistence in adults ranging from 2.58% to 6.76% [23]. ADHD is a disorder that is often multifactorial and can be identified in adolescence or adulthood. Although the DSM-5 [3] has raised the diagnostic age from 7 to 12 years, symptoms are often present much earlier [24]. Importantly, the longer the disorder is neglected, the more other behavioural, learning, and mood issues compound, making understanding the disorder, and thus the diagnostic evaluation, much more complex [25,26,27,28,29].

But what are the predictive signs that should not be underestimated? Can systematic observation of a child’s motor behaviours help educators and teachers recognise behavioural traits useful for the early identification of ADHD symptoms? Movement disorders, which are common among children with ADHD, particularly include disturbances in fundamental motor skills (FMSs). These disorders are characterized by low levels of fine and gross motor skills, difficulties with motor coordination, and incorrect posture, all of which may be related to delayed maturation of the prefrontal cortex [30,31,32]. 

The pre-school and school-age period is a particularly crucial phase for motor development, a complex process influenced by environmental and genetic factors throughout the entire life cycle. During their school years, children acquire FMSs, which include gross and fine motor skills. Gross motor skills (GMSs) involve movement of the entire body (the trunk and lower and upper limbs) in space and time, both freely and in relation to objects, whereas fine motor skills involve control of the hands and eyes, including fine manual control and hand–eye coordination [33].

The acquisition of adequate basic motor skills during the developmental years is critical as it influences sensory/perceptual and cognitive abilities, as well as social and emotional development [34,35]. GMSs, including locomotion and ball control skills, are cornerstones of child development and are fundamental skills not only for everyday tasks but also for active engagement in physical activities [36,37], excelling in sport-specific abilities, and ensuring successful participation in physical pursuits. Conversely, inadequate progress in motor development can affect motivation to engage in physical activity and encourage sedentary behaviours [38], thereby compromising not only motor development but also cognitive development, social interaction, and general health [39,40,41].

Additionally, some studies have found that children with ADHD and ASD struggle with age-appropriate FMSs. Therefore, more attention should be dedicated to teaching, practicing, and providing feedback in specific domains to promote the acquisition and competence of FMSs [42].

A meta-analysis investigated the effects of specific exercise interventions on FMSs in children with ADHD and ASD. The results showed that a specific physical exercise program (closed skills for 60 min, twice a week for 12 consecutive weeks) leads to improvements in both gross and fine motor skills in children with ADHD and those on the autism spectrum. This finding provides valuable insights for targeted interventions led by teachers, occupational therapists, special education teachers, physical education teachers, and parents of children with ADHD/ASD [43]. 

In particular, within the school context, the connection between physical activity and the enhancement of cognitive functions has been demonstrated [44,45]. Specifically, it has been observed that although physical activity can positively influence academic achievement, the presence of symptoms associated with ADHD has a detrimental effect on academic performance [46]. A recent review analysed several studies that implemented innovative embodied teaching approaches in the school context to promote cross-curricular learning through movement and corporeality in action and interaction. These learning environments emphasize the role of motor skills in enhancing overall learning [47].

Based on the points highlighted, monitoring gross motor development becomes crucial, especially in the school setting, as it provides valuable behavioural insights into the functioning and individual development of the child. In this study, the gross motor skills of a large sample of school-aged children were evaluated at school to assess whether the routine evaluation of gross motor skills could support clinicians in the timely detection of ADHD risk. The systematic observation of GMSs and their development may be an effective approach for understanding a child’s functioning and potential. It can also serve as a method for identifying specific indicators and drawing attention to critical aspects that require monitoring. This could contribute to enhancing both educational and clinical practices by facilitating coordination between schools and clinical structures for the early detection of neurodevelopmental disorders. The systematic observation of motor behaviour through standardised tests, along with observations during motor and sports activities, provides valuable information that helps to identify the child’s specific characteristics in terms of their strengths and weaknesses. These aspects are not only related to the motor domain but encompass a 360° observation that can help the educator/teacher understand the child’s potential for movement and functioning, as well as their intentions and motivations [48]. Given that motor performance is related to a wide range of developmental disorders and difficulties [49], and emerging technologies show promise in detecting neurodevelopmental disorders through movement patterns [50,51], establishing an association between gross motor skills and ADHD is fundamental in designing routine screening and diagnostic approaches. 

The objective of this research was to examine the relationship between gross motor development and ADHD symptoms in a cohort of Italian children. Specifically, we hypothesized that (a) there is an association between GMSs and symptoms of ADHD in school-aged children and (b) GMSs are associated with the risk of ADHD.

## 2. Materials and Methods

### 2.1. Participants

This study is part of a longitudinal project called “Benessere in Gioco” (BiG), which focuses on understanding motor and behavioural development among children in Northwest Italy. Written informed consent was obtained from parents or guardians, and verbal assent was received from the children. The ethical committee of the University of Turin approved this study (ID 100949). Employing a cross-sectional approach, data were collected from a substantial cohort of primary school children (N = 2677; mean age = 8.58 years, SD = 1.49 years, 51% males) identified through non-probabilistic sampling in schools in Northern Italy participating in this project. The inclusion and exclusion criteria are listed in Table 1. All the schools were public and had similar educational conditions.

### 2.2. Tools 

The Gross Motor Development-3 (TGMD-3) assessment tool [20] was utilised to measure the gross motor skills of the participants. The TGMD-3 evaluates fundamental gross motor skills across two categories: locomotor skills and ball skills [20,52,53,54]. Particularly, the TGMD-3 is used to measure the ways in which the child coordinates the globality of his or her body in an appropriate manner during some fundamental movements. It is a test focused more on the way in which movement is organised, rather than on assessment of the result or performance. In fact, the test does not evaluate running speed or the distance of a throw, but systematically observes the quality of motor action (coordination, fluidity, and self-organisation of movement). 

Gross motor skills were assessed based on the qualitative criteria for each TGMD-3 item as follows: every criterion was scored based on whether it was fulfilled (score awarded  =  1) or not (score awarded  =  0). Two trials were performed for each item, and the total score for each item was given by the sum of all the performance criteria scores in both trials. The sum of performance criteria scores from both trials was used to calculate the scores for the locomotor and ball control skill subtests, as well as for the overall TGMD-3 scores. Accordingly, the maximum score that a participant can obtain for their overall gross motor performance is 100, for the locomotor subtest is 46, and for the ball skill subtest is 54. The TGMD-3 was used because there is no gold standard for the assessment of gross motor skills [55]. The TGMD-3 is widely used globally for assessing these skills [56,57] and has demonstrated good psychometric properties [20,52,53,54].

In recent years, several validation studies of the TGMD-3 have been published [58,59,60,61], including an Italian version [20]. A recent review [62], through systematic analysis of the validity and reliability of assessment tools for the fundamental movement skills of school-age children, showed that thirty-four validation studies of the two versions of the TGMD have been published, providing evidence of validity and reliability ranging between good and excellent, confirming the effectiveness of the use of this tool in school settings.

All teachers completed the ADHD Rating Scale (SDAI) for each observed child, which is a standardized questionnaire that investigates various aspects of the child’s behaviour, as well as social and adaptive functioning. This scale consists of 18 items divided into two subscales (inattentiveness and impulsivity/hyperactivity), with a cutoff score of greater than 14 for each subscale. Each item is rated on a scale from 0 to 3, reflecting the frequency of observed behaviours, ranging from ‘never’ to ‘always’ [63]. The teachers were trained in the use of SDAI before the start of this study. It is necessary to point out that the SDAI scale adopted in our study has a screening function and is administered to teachers or an adult with knowledge of the child. In contrast to the complete assessment battery [63], which has a diagnostic purpose, the use of the scale for teachers alone has the sole aim of gathering first-hand information on the presence of the classic symptoms (presented by the DSM and ICD diagnostic manuals) of inattention and hyperactivity. This information serves a preventive function, aiding in acquiring a better understanding of the child’s behaviour. The parents of children who presented with high symptoms of ADHD were informed and referred to appropriate neuropsychiatric units or professionals in the area for further assessment when necessary. The TGDM-3 assessments and teachers’ reports were conducted once for each participant.

### 2.3. Statistical Analysis

The association between gross motor skills and the symptomatology of ADHD was investigated through multilevel modelling, incorporating random effects at a school level and considering demographics as covariates in the entire sample (N = 2677). The model was specified as follows: Total Score on the SDAI (dependent variable)~TGMD-3 Total Score (predictor) + demographics (age and gender) + (1|School). In the multilevel model, the children’s school was considered a random intercept. The Intraclass Correlation Coefficient (ICC) was calculated to address the between-school variance explained by different schools. Additionally, R^2^ was calculated to address the proportion of variance explained by the model fit. The model was specified considering that age and gender [64] are important variables reported in the literature related to gross motor skill performance, whereas the school was included as a random intercept to account for design effects. The homogeneity of variance between the schools was tested using an analysis of variance (F (15, 2661) = 0.17, *p* = 0.99), and the residuals were checked. Propensity score matching was performed to pair children at risk of ADHD with those not at risk (control group) in a 1:1 ratio, based on gender and age, to address any imbalance between the samples. Specifically, the propensity scores were estimated via logistic regression, considering age and gender as independent variables. The participants were then matched using nearest neighbour matching. A sensitivity analysis, conducted using Rosenbaum’s Sensitivity Test, was performed on the TGDM-3 Total Score. The analysis indicated that even when allowing for substantial bias in group assignments (Γ = 2), group differences persisted with a 95% confidence interval (upper bound = 0.001) [65]. Logistic regression was then performed to assess whether gross motor skills (TGMD-3 Total Score) are associated with the risk of ADHD. Additionally, multiple logistic regressions were performed to assess whether diverse types of gross motor skills (locomotor and ball skills) were associated with each subtype of ADHD (inattentive, hyperactive, and combined subtypes).

## 3. Results

**Figure 1:**
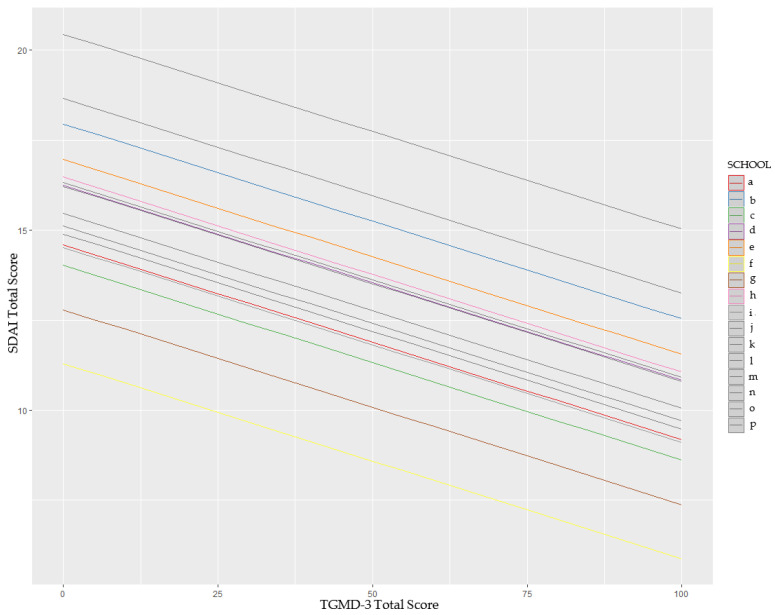
Multilevel model indicating the linear relation between TGMD-3 Total Score and SDAI Total Score.

The multilevel model for the entire sample indicated that gross motor skills are associated with the symptomatology reported using the SDAI, with higher proficiency in gross motor skills being associated with lower symptoms reported using the SDAI (B = −0.06, SE = 0.01, T = −4.05, R^2^ = 0.21, *p* < 0.001) (Figure 1).

Additionally, although age was not associated with the SDAI score, the model indicated that being a boy was associated with higher SDAI scores (B = −5.33, SE = 0.41, T = −12.80, *p* < 0.001). Participants’ different schools accounted for only 4% of the variance in the model (ICC = 0.04). Therefore, the subsequent analyses between participants at risk for ADHD and controls were conducted irrespective of school membership. For these analyses, 430 participants at risk were matched with 430 controls from the entire sample through propensity score matching. The descriptive statistics for participants matched according to age and gender are reported in Table 2. Logistic regression indicated that the Total Score of TGMD-3 was significantly associated with the risk of ADHD (B = −0.02, SE = 0.004, Exp(B) = 0.98, z = −3.03, *p* = 0.002). Considering the three different subtypes, locomotor skills were associated with the risk of the inattentive subtype (B = −0.03, SE = 0.01, Exp(B) = 0.97, z = −2.31, *p* = 0.021), whereas ball skills were not significant. Similar results were found for the combined subtypes. Locomotor skills were associated with this subtype (B = −0.04, SE = 0.01, Exp(B) = 0.96, z = −2.64, *p* = 0.008). The opposite was found for the hyperactive group, where locomotor skills were not significant but ball skills were significant (B = −0.04, SE = 0.01, Exp(B) = 0.96, z = −1.99, *p* = 0.046). These results are reported in Table 3.

## 4. Discussion

In this study, we analysed the association between gross motor skills and ADHD symptoms using a multilevel approach. The robust analysis, which involved a large sample of children, enabled pairing children at risk of ADHD with those not at risk (control group) in a 1:1 ratio, based on sex and age, to balance any disparities between the samples. The multilevel model revealed an association between gross motor skills and symptoms reported using the SDAI. Specifically, it emerged that greater competence in gross motor skills is associated with lower scores on ADHD symptom rating scales. This association suggests that higher levels of motor competence may have a potential protective effect against the development of ADHD symptoms, confirming the relationship between gross motor difficulties and ADHD [16,17]. To explore the relationships between gross motor development and ADHD symptoms more deeply, multiple logistic regression was conducted to determine whether the two subtests (locomotion and ball skills) were associated with individual ADHD symptoms (partial scale points for inattention, partial scores for hyperactivity, and total scores). Table 3 shows a significant relationship between inattention and locomotion skills, which is not evident with ball control skills. However, the symptom of hyperactivity is associated with the motor mastery of ball control skills, suggesting that a child who effectively coordinates the gross motor manipulation of objects might have a lower risk of developing ADHD symptoms. However, no association was found for locomotion skills. In the analysis between the total score and the two subtests, a positive relationship emerged between the risk of ADHD symptoms and locomotion skills, whereas ball control skills did not exhibit a significant association with the symptoms. These results demonstrate that a child’s behaviour varies based on the specificity of the most prevalent symptomatic trait. In general, locomotion skills exhibit a greater association with the scores recorded on the inattention scale and the combined total of the two scales, whereas ball control skills appear to be more closely associated with the hyperactivity scale. This difference could be partly explained by the distinct characteristics of the items in the two TGMD-3 subtests. Locomotion skills, which involve moving the body through space without manipulating objects, are simpler in terms of coordination. In contrast, the ball control subtest items require simultaneous management of body movements in relation to an object and an external target, demanding focused attentional resources. Therefore, locomotion skills require less attention and concentration, whereas ball control skills demand a higher level of attention, both for understanding task explanations (which can increase motivation) and for performing the tasks themselves. Indeed, a recent experimental study demonstrated the benefits of visuo-postural training for children with ADHD in improving motor control [66]. The study found that eye movements and postural stability improved through more effective use of the children’s attentional resources.

The adoption of an external, rather than internal, focus of attention was shown to be more effective in an experimental study comparing levels of motor learning in static balance among a sample of girls with ADHD. An external focus of attention decreased the children’s tendency to concentrate on themselves and, instead, encouraged them to focus more on achieving specific goals [67].

Although research in this specific area is limited, the existing studies provide valuable insights for understanding the importance of involving children with ADHD symptoms in tasks that require both gross and fine motor manipulation [68].

Several cross-sectional and longitudinal studies have demonstrated the close relationship between fine motor skills and achievement in school [69], as well as in reading and mathematics tests [70]. Fine motor skills in development appear to be closely related to intelligence in typically developing children between 4 and 11 years of age [71,72,73]. This highlights the importance of enhancing these skills during the early school years, particularly the need for continuous follow-up, allowing teachers to calibrate teaching actions by compensating for any delays or impairments in the learning and refinement of both fine and gross motor skills. One strength of this study is its large sample size. The population of children in pre-school and primary school in Northern Italy at the start of data collection was 928,938 [74]. The sample size required for a reliable representation of this population was 384 [75], which was achieved in the present study.

Despite the interesting results, this study has important limitations that must be considered. Firstly, the participants did not receive a confirmed clinical diagnosis, which means the results may be influenced by the lack of diagnostic specificity. Therefore, caution is recommended when generalizing these results. It is important to acknowledge that multi-informant reports could provide a more comprehensive assessment of ADHD symptoms. This study was based on teachers’ reports as children’s self-reports have lower validity than parents’ reports [76], and parents’ reports have the same accuracy as teacher reports [77]. Since the TGMD-3 is a standardised battery for gross motor skills, it is unlikely that considering another battery would have provided different results. In the present study, we did not consider balance as a possible associated skill. Therefore, future studies could consider this dimension. Additionally, the study utilised a cross-sectional experimental design; therefore, it would be valuable to replicate these findings using a longitudinal approach. Moreover, this study did not account for potential comorbid conditions related to ADHD. ADHD may co-occur with DCD as a comorbid neurodevelopmental disorder. Vulnerabilities associated with both disorders have an additive effect, resulting in a more severely impacted phenotype [78]. This implies more significant impairments in motor skills when both conditions are present [79]. The current study did not assess comorbidity with DCD due to the large sample of participants recruited, which represents a limitation. Future research should examine how both locomotor and object manipulation skills are affected by this comorbidity, as more severe impairments are expected. Analysing specific motor skills in the co-occurrence of ADHD and DCD could provide accurate information on how DCD affects different motor performances [80]. Further research should address this aspect. However, considering that different symptom subtypes indicated different associations with gross motor domains, it is reasonable to conclude that the observed difficulties are associated with ADHD symptoms rather than other conditions. Nevertheless, this study underscores the need for further research to better understand the relationship between ADHD and gross motor skills from a future perspective. For instance, although emerging technologies are not used in the current study, the established association between gross motor skills and ADHD is crucial for informing how they could be utilised in developing routine screening and diagnostic approaches.

## 5. Conclusions

ADHD, recognised as a neurodevelopmental disorder, impacts approximately 5% to 7% of the global child population [22]. The impact of ADHD extends to substantial economic burdens, as evidenced by significant expenditures in healthcare and education. For instance, in the Netherlands, expenditures are estimated at 1 billion euros [81], whereas in the USA, the financial impact ranges from 143 to 266 billion dollars [82] and in Australia the costs are estimated to be 12.76 billion dollars [83]. Beyond financial implications, ADHD profoundly impacts quality of life [84] and social acceptance [85] for affected children and adolescents. Consequently, timely identification and intervention regarding ADHD-related issues, including potential motor deficits, are imperative, irrespective of whether they stem solely from the symptoms of ADHD or are mediated by the presence of DCD [17] to prevent further problems in learning fine and gross motor skills, which are crucial to daily routines and academic performance [86].

The Test of Gross Motor Development-3 (TGMD-3) has emerged as a reliable assessment tool for evaluating gross motor skills among children with mental and behavioural disorders [53]. This study involved a large sample of school-aged children and aimed to identify an association between ADHD symptoms and general motor development. Although it did not establish a causal relationship between low TGMD-3 scores and ADHD symptoms, the effectiveness of the TGMD-3 in identifying critical aspects of motor development in school-age children, particularly those related to the possible risk of neurodevelopmental disorders, was demonstrated. The results indicate that lower competence in gross motor skills is associated with a higher risk of ADHD. Additionally, each subtype of ADHD appears to be associated with difficulties in different gross motor skill domains. This serves as a concern indicator for all stakeholders, including educators, parents, and physical education instructors, involved in the child’s developmental trajectory. Motor behaviour serves as a valuable indicator for evaluating broader developmental domains. Therefore, educational institutions and schools are encouraged to implement systematic monitoring protocols for evaluating general motor development. Such an approach not only facilitates the early detection of special educational needs but also promotes overall educational and well-being outcomes for children dealing with challenges related to ADHD.

## Figures and Tables

**Table 1:** Inclusion and Exclusion Criteria.

Inclusion Criteria	Exclusion Criteria
Age: between 5 and 11 years old	Children with certified disabilities
Children attending primary school classes (from the first to the fifth grade)	Children > 11 years old
Expression of informed consent from parents or guardians	Children whose parents have explicitly refused to participate in the research

**Table 2:** Participant groups after propensity score matching.

	Risk of ADHD	Control Group	Test Statistic	*p*
N	430	430		
Mean Age (SD)	8.51 (1.44)	8.50 (1.44)	T = 0.10	0.92
Gender (M; F)	340; 126	304; 126	ꭓ^2^ = 0.00	1.00

**Table 3:** Results from multiple logistic regression on different subtypes of ADHD risk.

	B	SE	Exp(B)	Z	*p*
*Inattentive*					
Intercept	−0.73	0.59	−	−1.22	0.219
Locomotor Skills	−0.03	0.01	0.97	−2.31	0.021
Ball Skills	0.00	0.01	1.00	0.727	0.467
*Hyperactive*					
Intercept	−1.99	0.92	−	−2.03	0.042
Locomotor Skills	0.01	0.01	1.01	0.424	0.671
Ball Skills	−0.04	0.01	0.96	−1.99	0.046
*Combined*					
Intercept	−0.03	0.38	-	−0.073	0.937
Locomotor Skills	−0.04	0.01	0.96	−2.64	0.008
Ball Skills	0.00	0.01	1.00	0.27	0.78

## Data Availability

The data presented in this study are available on request from the corresponding author. The data are not publicly available due to privacy reasons.
